# Aging in Democracy: Accomplishments and Challenges of Long-Term Care Policy in Post-Authoritarian Taiwan

**DOI:** 10.1093/geroni/igaf122.1904

**Published:** 2025-12-31

**Authors:** Mo’e Yaisikana

**Affiliations:** University of California, Berkeley, Berkeley, California, United States

## Abstract

Taiwan has one of the fastest-growing aging populations in the world. As a rapidly aging society, the Taiwan government implemented a national universal Long-Term Care (LTC) service program in the early 2000s. The enactment of the Long-Term Care Services Act in 2017 was a milestone in further institutionalizing LTC into a centralized delivery network. The democratic political system played a crucial role in establishing LTC through legislation and parliamentary scrutiny. This paper investigates how democratic politics contributes to the accomplishments of elder care policy in the recently democratized Taiwan with archival data and ethnography conducted in rural villages in an extended case study approach. Findings suggest that older adults and their needs comprised a nascent social identity with unique interests that merge elders, caregivers, and business owners in the aging society. Amplified through their population size in multi-level electorate participation, they further propelled the advocacy for national-level policies and public service. Nevertheless, without a robust social insurance system, the tax-based governmental budget implied the financial insecurity and solid regulations of LTC. These regulations created incompatibilities between the local knowledge of caregiving and public services administration that may inhibit service development. The democratization established channels for private interests to be institutionalized, bridging a bottom-up path for welfare development, but it also depoliticizes elders’ care in administration and turns older adults into the governable population. Localization and diversification have become LTC’s following goals in Taiwan. This case study offers critical insights for developing sustainable care policies in aging democratic states worldwide.

